# A Systematic Review Concerning the Relation between the Sympathetic Nervous System and Heart Failure with Preserved Left Ventricular Ejection Fraction

**DOI:** 10.1371/journal.pone.0117332

**Published:** 2015-02-06

**Authors:** Willemien L. Verloop, Martine M. A. Beeftink, Bernadet T. Santema, Michiel L. Bots, Peter J. Blankestijn, Maarten J. Cramer, Pieter A. Doevendans, Michiel Voskuil

**Affiliations:** 1 Department of Cardiology, University Medical Center Utrecht, Utrecht, the Netherlands; 2 The Julius Center for Health Sciences and Primary Care, University Medical Center Utrecht, Utrecht, the Netherlands; 3 Department of Nephrology, University Medical Center Utrecht, Utrecht, the Netherlands; Loyola University Chicago, UNITED STATES

## Abstract

**Background:**

Heart failure with preserved left ventricular ejection fraction (HFPEF) affects about half of all patients diagnosed with heart failure. The pathophysiological aspect of this complex disease state has been extensively explored, yet it is still not fully understood. Since the sympathetic nervous system is related to the development of systolic HF, we hypothesized that an increased sympathetic nerve activation (SNA) is also related to the development of HFPEF. This review summarizes the available literature regarding the relation between HFPEF and SNA.

**Methods and Results:**

Electronic databases and reference lists through April 2014 were searched resulting in 7722 unique articles. Three authors independently evaluated citation titles and abstracts, resulting in 77 articles reporting about the role of the sympathetic nervous system and HFPEF. Of these 77 articles, 15 were included for critical appraisal: 6 animal and 9 human studies. Based on the critical appraisal, we selected 9 articles (3 animal, 6 human) for further analysis. In all the animal studies, isoproterenol was administered to mimic an increased sympathetic activity. In human studies, different modalities for assessment of sympathetic activity were used. The studies selected for further evaluation reported a clear relation between HFPEF and SNA.

**Conclusion:**

Current literature confirms a relation between increased SNA and HFPEF. However, current literature is not able to distinguish whether enhanced SNA results in HFPEF, or HFPEF results in enhanced SNA. The most likely setting is a vicious circle in which HFPEF and SNA sustain each other.

## Introduction

Heart failure with preserved left ventricular ejection fraction (HFPEF) affects about half of all patients with a clinical presentation of heart failure (HF).[1,2] There is no consensus concerning the definition of HFPEF. The European guidelines define HFPEF as a clinical syndrome in which classical HF symptoms are present, accompanied by a normal or only mildly reduced left ventricular (LV) systolic function.[3] Using this definition, HFPEF becomes a mixed collection of different underlying causes of HF. The American Heart Association (AHA) guidelines and the consensus statement of the European Society of Cardiology (ESC) define HFPEF as a clinical HF state, which is accompanied by objective evidence of diastolic dysfunction (DD).[4,5]

Irrespective of the definition, we still have much to learn about HFPEF. This is all the more important since no successful treatment is available yet.[3,4] A number of studies have been conducted investigating different pharmacological treatment strategies for HFPEF. Unfortunately, these studies failed to provide unambiguous results.[6–10]

Even though HFPEF has been the focus of various mechanistic studies, the exact pathophysiology is still unknown.[11] It is generally accepted that HFPEF is characterized by prolonged isovolumic LV relaxation, slow LV filling, and an increased diastolic LV stiffness.[12] The consequent impairment of diastolic filling leads to an inappropriate pressure increase after volume load.[13] Eventually, this may lead to heart failure.[14,15] The sympathetic nervous system (SNS) may play an important role in the genesis of HFPEF when accompanied by DD.[16] The underlying structural changes in the myocardium seen in HFPEF include the same spectrum of changes associated with catecholamine-induced cardiomyopathies.[17,18]

However, while the role of the increased sympathetic nerve activity (SNA) in the development and progression of HF with reduced ejection fraction (HFREF) is well established[19,20], to our knowledge, no systematic review has yet evaluated the relationship between SNA and HFPEF. Therefore, the objective was to systematically evaluate the role of SNA in HFPEF. In this respect, only HFPEF in combination with DD is taken into account.

The activity of the SNS can be measured in different ways. Examples are measurement of plasma or urinary norepinephrine (NE) level, assessment of local NE spillover, muscle sympathetic nerve activity (MSNA), iodine 123-metaiodobenzylguanidine (MIBG), or heart rate variability (HRV).

## Methods

### Search strategy

This systematic review was conducted and reported in accordance with the “preferred reporting items for systematic reviews and meta-analyses” (PRISMA) statement.[21] We conducted a systematic review to determine if there is a relationship between the sympathetic nervous system and heart failure with preserved LVEF. All available literature in the PubMed, Embase and Cochrane databases was searched using a pre-defined search strategy (S1 Appendix). A librarian checked the syntax before the search was conducted. The titles and abstracts of the retrieved articles were reviewed by three authors (WLV, MMAB, BTS). Full-text papers were retrieved from abstracts selected for further review. The references of these papers were also reviewed to identify relevant articles that may have been missed by the search strategy, e.g. studies that were not found due to negative results. If necessary, individual researchers were contacted by e-mail to obtain the full text, or to enquire about unpublished or unreported results.

All full-text articles were reviewed by 3 authors (WLV, MMAB, BTS) using pre-defined inclusion/exclusion criteria (Table 1). Articles were only included when the inclusion criteria of HFPEF were clearly defined. Only articles that included DD in the definition of HFPEF were included, studies about HFPEF based on valvular dysfunction or other disease entities were excluded. Citations from journals in languages other than English were not included. No pre-specified limitations were placed regarding species (human or animal) or NYHA functional class. Individual case reports, editorials, expert opinions, and review articles were excluded, as were studies regarding the diagnosis or treatment of HFPEF. Studies investigating the prognosis of patients with HFPEF were also excluded. To minimize the risk of multiple publication bias, we only included publications with a pathophysiological objective when they contained original data. We pre-specified the data to be extracted from the included studies before reading the articles. Two authors (WLV, MMAB) independently extracted these data and listed them in a table.

**Table 1:** Pre-set inclusion and exclusion criteria.

Inclusion criteria	Exclusion criteria
Investigating the relationship between HFPEF and the sympathetic nervous system	Investigating systolic heart failure
Investigating the relationship between diastolic dysfunction and the sympathetic nervous system	Only investigating LVH without giving information about diastolic dysfunction
	No original data (i.e. review, expert opinion)
	Study does not investigate relation SNA and HF
	Only abstract
	Full text in language other than English
	Therapeutic study
	HFPEF based on valve dysfunction, myocardial ischemia or hypertrophic cardiomyopathy
	Prognostic study

### Critical appraisal and analysis

Critical appraisal was independently performed using pre-set criteria. These criteria are outlined in Table 2 and Table 3. In advance we decided only to include an article in the final analysis if it scored at least half of the maximum available points. Since we expected that there would be a large diversity in outcome measures, no pre-defined principal summary measures were composed. Studies were graded for the modality used to measure sympathetic activity, as reliability of these modalities differ.[22,23] Local NE spillover, MSNA, and MIBG are considered as the best and most direct measures of sympathetic activity.[24,25] Serum levels of NE and HRV are an indirect measure of SNA and are considered as less qualitative measures of sympathetic activity.[23–25]

**Table 2:** Critical appraisal of animal studies.

First author, year	Study aim	Clearly defined hypothesis	Model to induce HFPEF	Assessment of diastolic dysfunction	Assessment of sympathetic activity	Clear report of findings	Value of study	Score
Grimm, 1998	+	+/-	+	+	+/-	+/-	+	4
Krishnamurthy, 2007	-	+/-	+	+	+/-	+/-	+/-	1
LaCroix, 2008	-	+	+	+	+/-	+	-	2
Brooks, 2009	+	+/-	+	+	+/-	+	+/-	4
Ma, 2011	-	+/-	+	+	+/-	+/-	+/-	1
Yoshikawa, 2012	+	+	+	+	+/-	+/-	+/-	4

**Study aim**:+: study is focused on interpreting the relation between sympathetic activity (SNA) and diastolic dysfunction (DD); -: study is not focused on interpreting the relation between SNA and DD. **Clearly defined hypothesis**: +: hypothesis clearly defined; +/-: aim of study clearly defined, no hypothesis formulated; -: no clear aim nor hypothesis. **Model to induce HFPEF**: +: ISO infusion; -: transaortic constriction. **Assessment of diastolic dysfunction**: +: invasive measurement of LV diastolic filling pressures or echocardiographic evaluation of DD according to latest ESC guidelines; +/-: echocardiographic evaluation without use of E/E’; -: confirmation of normal LVEF only. **Assessment of sympathetic activity**: +: yes; +/-: no. **Clear report of findings**: +: results clearly described AND critical about own research; +/-: results clearly described OR critical about own research; -: results not clearly described AND not critical about own research. **Value of study**: To what extent is the study relevant to answering the current question.

The score displayed in the right column is the sum of scores: “+” accounts for 1 point; “+/-” for 0 points; “-” for -1 point.

**Table 3:** Critical appraisal of human studies.

First author, year	Study design	Number of patients	Study aim	Clearly defined study aim	Patient selection	Assessment of diastolic dysfunction	Assessment of sympathetic activity	Clear report of findings	Value of study	Score
Nixdorff, 1997	Cohort	10	++	+	N/A	+/-	+/-	+	+/-	4
Hirono, 2001	Cohort	26	-	-	+	+/-	+/-	+	-	-1
Vinch, 2003	Cross-sectional	14	-	+	+	+/-	-	+/-	-	-1
Arora, 2004	Cross-sectional	19	++	+	+	-	+/-	+	+	5
Piccirillo, 2006	Cross-sectional	30	++	+	+	+/-	+/-	+	+	6
Sugiura, 2006	Cohort	34	+	+	+	+/-	+	+	+	6
Tsuchida, 2007	Cross-sectional	8	-	+	+	-	+	+	-	1
Grassi, 2009	Cross-sectional	17	+	+	+	+/-	+	+	+	6
deSouza, 2013	Cross-sectional	15	+	+	+	+	+	+	+	7

**Number of patients** = : Number of patients with diastolic dysfunction/HFPEF. **Study aim**: ++: study is focused on interpreting the relation between SNA and DD **AND** patient selection was clearly explained (diastolic dysfunction defined and not just distinction between LVEF </> 45%) **AND** data collection was clear. +: study is focused on interpreting the relation between SNA and DD **AND** patient selection was clearly explained **OR** data collection was clear; -: study is focused on interpreting the relation between SNA and DD **OR** patient selection was clearly explained **OR** data collection was clear; —: study is not focused on interpreting the relation between SNA and DD **AND/OR** patient selection was not clearly explained **AND/OR** data collection was not clear. **Patient selection**: +: Sole HFPEF or clear distinction between HFPEF and HFREF; -: no clear distinction between HFPEF and HFREF. **Assessment of diastolic dysfunction**: +: invasive measurement of LV diastolic filling pressures OR echocardiographic evaluation of DD according to latest ESC guidelines; +/-: echocardiographic evaluation without use of E/E’; -: confirmation of normal LVEF only. **Evaluation of sympathetic activity**: ++: NE-spillover locally measured; +: MSNA OR MIBG; +/-: HRV or adrenergic stimulation; -: plasma NE concentration. **Clear report of findings**: +: results clearly described AND critical about own research; +/-: results clearly described OR critical about own research; -: results not clearly described AND not critical about own research. **Value of study**: To what extent is the study relevant to answering the current question.

The score displayed in the right column is the sum of scores: “+” accounts for 1 point; “+/-” for 0 points; “-” for -1 point.

## Results

**Fig 1:**
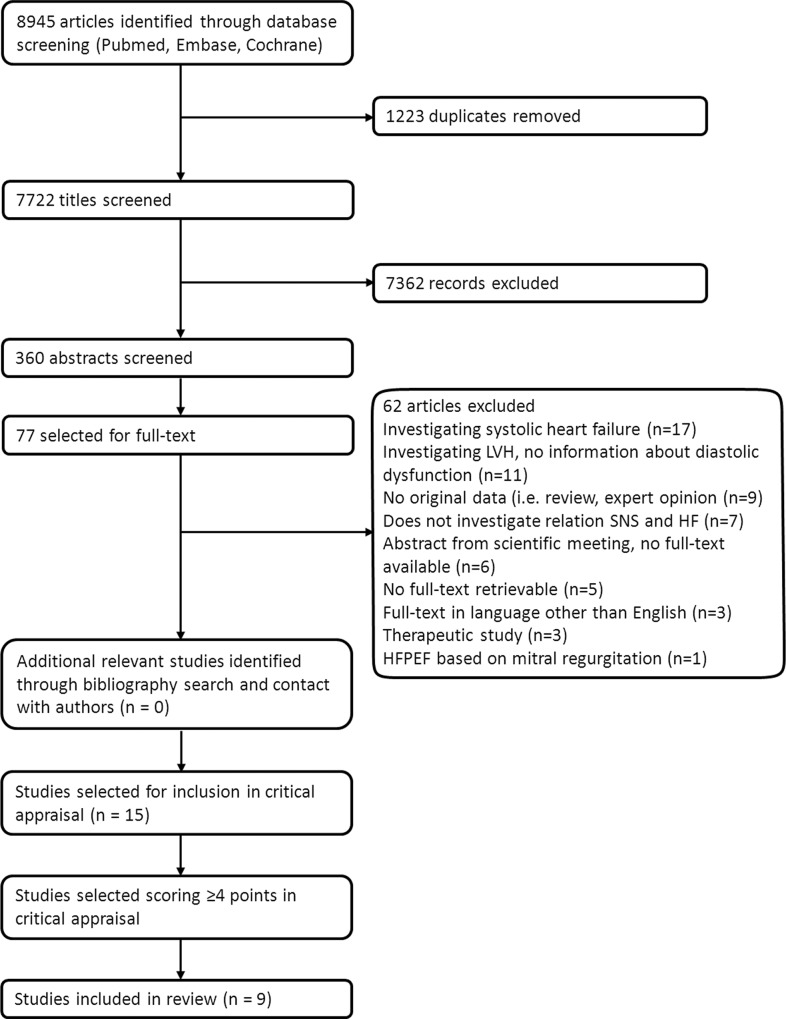
Flowchart of the search.

The search was conducted on October 31st, 2013 and identified 7722 unique articles. The search was updated on April 10^th^, 2014. A flowchart of the search is depicted in Fig. 1. After screening the titles and abstracts 77 articles remained that met the criteria for full text review. Fifteen full-text articles (6 animal studies; 9 human) were considered relevant to the study and were included in the critical appraisal.[26–40] A summary of these 15 studies is outlined in S2 Appendix.

### Animal studies

The animal studies that met the criteria for critical appraisal investigated whether a change in activity of the sympathetic nervous system might induce diastolic dysfunction. All animal studies used a model of isoproterenol (ISO) to mimic an increased SNA. Isoproterenol is a non-selective β-adrenergic agonist structurally similar to epinephrine. The assumed increase in SNA was not measured in any of the animal studies.

Three animal studies scored high (4 points or more) in the critical appraisal and were therefore further evaluated.[26,27,31] Rejected studies scored less than half of the available points mainly because the study-aim was insufficiently focused on DD or HFPEF.

In brief, the three selected animal studies demonstrated that administration of a β-adrenergic agonist established diastolic dysfunction in an experimental setting: the first study is the study from Grimm et al. Authors observed that dosages up to 150 mg/kg ISO led to diastolic dysfunction in mice as evaluated by echocardiography.[27] Dosages higher than 150 mg/kg led to systolic heart failure or death.[27] In the second study: the study of Brooks et al. an abnormal LV diastolic pressure-volume (PV) relationship and an increased myocardial stiffness in mice treated with 10 mg/kg ISO for 5 days was observed without impairment of systolic function.[26] The third study: the study of Yoshikawa et al. treated rats with 2.4 mg/kg/day ISO for 7 days and observed a significant increase in fibrosis, accompanied by an increase in LV hypertrophy by histology and a decrease in diastolic function by echocardiography.[31]

A major drawback of these three studies is that the percentage of animals in which ISO administration did or did not lead to DD or HFPEF (‘responder rate’) was not clearly reported. Impairment of DD was already observed after a dosage of 2.4 mg/kg/day ISO for 7 days. A single dosage of 150 mg/kg ISO also induced DD. Higher dosages led to systolic heart failure.

### Human studies

No studies could be found that prospectively studied a human cohort whether an increased SNA leads to HFPEF during follow-up. In most human studies evaluated by the critical appraisal, patients were included that already had DD based on echocardiographic data. Therefore, the natural progression to HFPEF could not be studied.

All studies were hindered by a small sample size of patients (max. 34 patients). Another limitation is that not all studies reported the diastolic parameters: Sugiura and Arora et al. included HF-patients with a good ventricular function (LVEF>45%) irrespective of diastolic parameters.[32,38] Echocardiographic examination was often limited to E/A ratio and deceleration time, despite that E/e’ is nowadays considered a more reliable parameter.[5] An explanation may be the evolving guidelines on assessment of diastolic function that did not involve E/e’ at the time the first studies were conducted. For the assessment of SNA, different entities were used in the human studies (i.e. heart rate variability (HRV), MSNA, MIBG, or norepinephrine levels). Six out of nine human studies scored 4 points or more in the critical appraisal and were therefore further evaluated.

In brief; the 6 human studies selected for further evaluation confirmed a relationship between SNA and HFPEF. Arora et al. observed that patients with HFPEF exhibit a reduction in HRV compared to control subjects.[32] Patients with HFREF had more decreased HRV values compared to patients with HFPEF. In the study of Grassi et al. abnormal baroreflex modulation and increased MSNA-levels were observed in hypertensive patients with DD compared to hypertensives without DD and normal controls.[33] Nixdorff et al. observed an increase of peak early (E-wave) and late (A-wave) diastolic filling velocities and a shortening of deceleration time after administration of even the lowest dose of ISO (0.1 ug/min).[35] Piccirillo et al. performed HRV and observed that hypertensives with diastolic dysfunction have a higher sympathetic and lower vagal modulation of the sinus node compared to hypertensives without DD and normotensive controls.[36] deSouza et al. observed that patients with HFPEF had higher MSNA values compared to hypertensive patients with normal diastolic function although HRV values were similar among the groups.[37] In the study of Sugiura et al. it was concluded that cardiac SNA as assessed by MIBG increases proportionally with severity of HFPEF.[38]

## Discussion

To our knowledge, this is the first systematic review investigating the relationship between sympathetic nerve activity and HFPEF in combination with DD. Based on the animal studies we concluded that administration of ISO and therefore increased SNA is related to HFPEF in an experimental setting. Based on the human studies, we concluded that an increased SNA—irrespective of the method of assessment—is indeed related to diastolic dysfunction and/or true HFPEF. However, the available literature about this topic is very scarce, let alone that results could be pooled. Moreover, administration of ISO in animals is not exactly the same as the increase in SNA in humans. However, no animal model to induce HFPEF has been accepted so far and we consider ISO administration the best available at present.

As mentioned above, different definitions are used for HFPEF.[3,5] In the current review, the recommendations from the AHA guidelines and the ESC consensus document were followed, thereby excluding HFPEF based on hypertrophic cardiomyopathy among others.[4,5] We chose to follow this line in order to obtain a set of studies with a more or less homogeneous patient population.

In the human studies, different ways of assessing sympathetic activity were used. The most straightforward method to measure SNS is measurement of plasma or urinary (NE) level. Plasma NE concentrations however are a resultant of removal rates and not selectively release rates.[41] Also, the precise origin of urinary NE levels is a matter of debate. Assessment of local NE spillover by a radiotracer technology displays the rate at which NE is released from the sympathetic nerves into the circulation. This is quantified by intravenous infusion of titrated NE combined with regional sampling. MSNA is a real-time measure of sympathetic nerve activity. Multiunit recordings of efferent postganglionic MSNA are obtained with a tungsten microelectrode into a muscle fascicle of the peroneal nerve.[42] MIBG imaging uses a norepinephrine analogue labeled with a radioactive isotope to image adrenergic receptors in many organs, including the heart.[43] MIBG imaging has been shown a very reliable marker of sympathetic activity in both HFPEF and HFREF disease states.[44] Moreover, Nakata et al demonstrated the long-term prognostic value of altered cardiac sympathetic function as assessed by MIBG imaging in HF patients.[45] HRV displays the variability of the resting heart rate and is a measure of the balance between the sympathetic- and parasympathetic nervous system.[46] HRV is linked through the baroreceptor reflex and a more indirect way to measure SNA.[23] Not all ways of SNA measurement are even reliable. This supposed difference in reliability was taken into account in the critical appraisal. Although NE-spillover and MSNA are among the most reliable ways to measure SNA, they are semi-invasive and time-consuming. Moreover, MSNA is hard to obtain in small animals like mice.[25] MIBG washout rate has a strong correlation with MSNA and therefore allows non-invasive assessment of general sympathetic nerve activity.[24] In the studies selected for critical appraisal, local NE spillover was not used as a method to quantify sympathetic activity.

The study of Grassi et al. showed conflicting results; no difference in plasma NE-concentration was observed whereas MSNA values were altered in the patients with DD.[33] In our analysis, the results of MSNA were taken into account whereas the NE-results were not due to the limited sensitivity of these markers of sympathetic tone. In the studies of Arora et al. and Piccirillo et al., HRV was assessed to obtain information about the parasympathetic nervous system.[32,36] In the critical appraisal these studies scored less due to the use of HRV. Since HRV is an indirect measurement of SNA, it is not known whether the results of Arora et al. may lead to an over- or underestimation of the relation between HFPEF and SNA.[23]

### Can the results be explained by more diseased states?

From a critical point of view, some experts argued that the increased SNA in patients with DD can be attributed to the higher BP since that is often present in patients with DD.[47] To respond to this criticism, Grassi et al. and Piccirillo et al. only included patients with similar blood pressure (BP) levels.[33,36] Based on these 2 studies, we argue that increased sympathetic activation seen in DD is not attributable to a more diseased hypertensive state.[33,36] As left ventricular hypertrophy (LVH) is also related to an increased SNA and often present in DD, the presence of LVH may have caused us to overestimate the increased SNA.[48] Yet Grassi et al. found similar LV masses among both hypertensive groups (with and without DD).[33]

Since β-adrenergic stimulation often causes both HFPEF and HFREF, SNS-induced HFPEF could be a precursor state of HFREF or both diseases could be entities resulting from increased SNA.[49] The study of Grimm et al. contributes to this discussion by showing systolic HF after administration of higher dosages of ISO.[27] Seeland et al. described comparable results in a mouse model undergoing adrenergic stimulation: five months after stimulation LVH was observed in all mice.[50] However, 12 months after stimulation ventricular dilatation and accompanying systolic dysfunction was observed in all mice.[50] More recent studies, however, have made clear that the two diseases are indeed two separate entities.[49,51]

### Diastolic dysfunction and SNA; the chicken or the egg?

As Rosendorff previously discussed, it is not yet clear whether DD potentiates the sympathetic activation or whether the increased SNA causes DD.[47] Based on their results, Grassi et al. concluded that DD enhances the already elevated MSNA.[33] However, the authors admitted that their data did not allow them to determine whether the greater SNA observed in patients with DD is the cause or the consequence of the cardiac alteration.[33] Other studies included in the current review concluded that it should be the other way around.[26,35] Moreover, sympathico-inhibition has shown to delay the progression of DD.[52–55] Finally, Leite-Moreira et al. showed that β-adrenergic stimulation influences cyclic AMP, resulting in a changed diastolic relaxation.[56] Rosendorff set out 2 plausible ways that increased SNA causes DD: an indirect and a direct way. In the indirect way SNA induces hypertension, which imposes a mechanical load on the LV and consequent stiffening of the ventricles.[47] In the direct way, SNA has a direct effect on both hypertension and diastolic dysfunction.[47] Increased SNA activity plays a role in cardiac remodeling.[57] This is illustrated by the fact that sympathetic stimulation can induce pro-inflammatory cytokine expression[58] and can induce alterations in the sarcoplasmic reticulum, plasma membrane, and cytoskeletal proteins.[12,56] However, one crucial question cannot be answered with the above theories: what is the trigger for an increased SNA? A mechanism is needed that is responsible for SNS stimulation. In HF*R*EF an ischemic model can be the precursor of increased SNA.[59] However, in HFPEF ischemia is often not present as a precursor of SNA.[4]

With the current available evidence, we cannot simply state that one causes the other. Paulus et al. has clearly set out that different comorbidities contribute to a systemic inflammatory state, which induces oxidative stress in the coronary microvascular endothelium. [60] The presence of reactive oxygen species (ROS) is important in the paradigm proposed by Paulus et al.[60] ROS are strongly believed to be related to increased SNA.[61,62] We believe that a vicious circle is present in which HFPEF and SNA sustain each other. It is highly likely that other factors like the metabolic syndrome or renal ischemia are triggers for this circle.[63]

### The effect of sympathico-inhibition on HFPEF and DD

Until now, no treatment has yet convincingly shown to improve clinical status, morbidity and mortality in HFPEF.[6,64] It is of relevance whether therapies targeting the sympathetic nervous system are successful in HFPEF. Based on their sympatholytic effect, beta-blockers may be useful in HFPEF. The SENIORS trial suggested that nebivolol may be beneficial in elderly patients with HFPEF.[65] As a derivative of SNA, angiotensin II may be a target for treatment. While RAS inhibition has been shown to reduce SNA,[66] the CHARM study found no clear benefit in patients with HFPEF treated by RAS inhibition.[67] Varying results were reported in other studies investigating the effect of RAS-inhibition in patients with HFPEF: in a meta-analysis, RAS-inhibition was not associated with consistent reduction in HF hospitalization or mortality in HFPEF-patients.[68]

Echocardiography is a reliable tool to objectively assess diastolic function. The effects on echocardiography should be taken into account when conducting a therapeutic study in a HFPEF population. Echocardiographic parameters have been used in some studies investigating aldosterone antagonists, beta-blockade, exercise training, and RAS-inhibition. Although some studies reported an improvement in clinical state, no clear effects on echocardiographic parameters were observed.[10,69,70] Based on our results, it is plausible that modulation of SNA can improve the clinical status of patients with HFPEF. Unfortunately, no studies investigating a treatment for HFPEF have evaluated SNA after treatment. One study of interest is that of Brandt et al. who recently showed an improvement in diastolic function after renal denervation.[71] The authors established a decrease in sympathetic activity by renal denervation and consequently observed improvement in echocardiographic parameters.[71] Therefore, renal denervation may be an attractive option for the treatment of HFPEF by disrupting the vicious circle between HFPEF and hyperactive SNA.

### Limitations of the current review

First of all, the available evidence is limited and heterogeneous in design. This heterogeneity may have influenced our results. We tried to uniform the different studies by using the critical appraisal.

Although our search strategy was extensive and also focused on studies that showed HFPEF and SNA to be unrelated, it is possible that our search resulted in a relative over-representation of positive studies. By checking references of selected articles we tried to obtain studies that reported negative findings about the suggested relation. This search did not result in any relevant full text articles.

In the human studies, we could not report about medical history, medication use and other determinants for heart failure because these determinants were not clearly reported. It should, however, be taken into account that most patients studied were treated with drugs that affect sympathetic activity. Since most antihypertensive drugs (indirectly) lower SNA[72], the currently observed relation between SNA and HFPEF may be an underestimation. Potentially, an even stronger association between SNA and HFPEF does exist.

Not all human studies included patients with HFPEF; half of the human papers studied patients with DD instead of HFPEF. However, DD is an important, if not the main, precursor of HFPEF. Moreover, Sugiura et al. showed a higher cardiac sympathetic activity in HFPEF patients with a higher NYHA class.[38] Though, at this point we should keep in mind that the clinical symptoms in HFPEF are not solely explained by DD, but can also be explained by reduced chronotropic, vasodilator, and cardiac output reserve during exercise.[73]

A possible confounding factor in this review is that HFPEF-patients are more often older. Age itself is related to sympathetic activity and may be a confounder in the available literature.[4] Selected studies did not report whether they corrected for the baseline characteristics. To complicate matters, HFPEF has only recently been recognized as an important clinical problem and preserved ejection fraction was previously often considered as a diagnosis of exclusion.[16] Therefore the available literature is less extensive compared to HFREF.

## Conclusion

Based on our results, we conclude that current literature confirms a relation between increased SNA and HFPEF. However, current literature is not able to distinguish whether enhanced SNA results in HFPEF, or HFPEF results in enhanced SNA. The most likely setting is a vicious circle in which HFPEF and SNA sustain each other. Disruption of this vicious circle may be an attractive treatment option.

## Supporting Information

S1 Checklist PRISMA checklist. (DOC)

S1 Appendix The syntax used as search strategy. (DOCX)

S2 Appendix Summary of included studies in the review. (DOCX)
