# Supplementary material for: A Systematic Review Concerning the Relation between the Sympathetic Nervous System and Heart Failure with Preserved Left Ventricular Ejection Fraction
Source: PLoS One. 2015 Feb 6;10(2):e0117332. doi: 10.1371/journal.pone.0117332 (PMC4319815; doi:10.1371/journal.pone.0117332)
Supplement: S1 Appendix — (DOCX) [file pone.0117332.s002.docx]

**Appendix S1: syntax**

**PUBMED**

((((((((((((((((LV end diastolic volume[Title/Abstract] OR LV end diastolic pressure[Title/Abstract]) OR left ventricle end diastolic pressure[Title/Abstract]) OR LVEDP[Title/Abstract]) OR left ventricle end diastolic volume[Title/Abstract]) OR LV wall thickness[Title/Abstract]) OR left atrial volume[Title/Abstract]) OR left ventricle mass[Title/Abstract]) OR left ventricle volume[Title/Abstract]) OR LV mass[Title/Abstract]) OR LV volume[Title/Abstract]) OR LA volume[Title/Abstract]) OR LVEDV[Title/Abstract]) OR heart ejection fraction[Title/Abstract]) OR diastole[Title/Abstract]) OR hypertrophy left ventricular[Title/Abstract]) OR ("hfnef"[Title/Abstract] OR "hfpef"[Title/Abstract] OR "diastolic heart failure"[Title/Abstract] OR ("diastolic"[Title/Abstract] AND "heart"[Title/Abstract] AND "failure"[Title/Abstract]) OR ("heart"[Title/Abstract] AND "failure"[Title/Abstract] AND "normal"[Title/Abstract] AND "ejection"[Title/Abstract] AND fraction[Title/Abstract]) OR ("heart"[Title/Abstract] AND "failure"[Title/Abstract] AND "normal"[Title/Abstract] AND ("lvef"[Title/Abstract] OR "ef"[Title/Abstract])) OR ("heart"[Title/Abstract] AND "failure"[Title/Abstract] AND "ejection"[Title/Abstract] AND fraction[Title/Abstract] AND "preserved"[Title/Abstract]) OR ("heart"[Title/Abstract] AND "failure"[Title/Abstract] AND preserved[Title/Abstract] AND ("lvef"[Title/Abstract] OR "ef"[Title/Abstract])) OR (("normal"[Title/Abstract] OR "preserved"[Title/Abstract]) AND ("ventricle"[Title/Abstract] OR "ventricular"[Title/Abstract]) AND ("heart"[Title/Abstract] AND "failure"[Title/Abstract] AND "function"[Title/Abstract])) OR ("diastolic"[Title/Abstract] AND "dysfunction"[Title/Abstract]) OR ("diastolic"[Title/Abstract] AND "heart"[Title/Abstract] AND "disease"[Title/Abstract]))) AND (((("sympathetic"[Title/Abstract] AND nerve[Title/Abstract] AND "system"[Title/Abstract]) OR ("sympathetic"[All Fields] AND "nervous"[Title/Abstract] AND "system"[Title/Abstract]) OR ("sympathetic"[Title/Abstract] AND "activity"[Title/Abstract]) OR ("sympathic"[Title/Abstract] AND "activity"[Title/Abstract]) OR ("sympathetic"[Title/Abstract] AND "nerve"[Title/Abstract] AND "activity"[Title/Abstract]) OR "sympathicus"[Title/Abstract] OR ("orthosympathetic"[Title/Abstract] AND "activity"[Title/Abstract]) OR ("orthosympathetic"[Title/Abstract] AND "nervous"[Title/Abstract] AND "system"[Title/Abstract]) OR ("orthosympathetic"[Title/Abstract] AND nerve[Title/Abstract] AND "system"[Title/Abstract]) OR ("sympathetic"[Title/Abstract] AND ("nerve"[Title/Abstract] OR "nerves"[Title/Abstract])) OR "sns"[Title/Abstract]) OR (((((((((((((((norepinephrine[Title/Abstract] OR noradrenaline[Title/Abstract]) OR noradrenalin[Title/Abstract]) OR adrenalin[Title/Abstract]) OR adrenaline[Title/Abstract]) OR epinephrine[Title/Abstract]) OR ("catecholamine"[Title/Abstract] OR "catecholamines"[Title/Abstract])) OR (isoproterenol[Title/Abstract] OR ISO[Title/Abstract])) OR ((reactive[Title/Abstract] AND oxygen[Title/Abstract]) AND species[Title/Abstract])) OR (("microneurography"[Title/Abstract] OR peroneal nerve[Title/Abstract]) OR "microneurographic"[Title/Abstract])) OR "noradrenergic"[Title/Abstract]) OR (autonomic[Title/Abstract] AND stimulation[Title/Abstract])) OR (autonomic[Title/Abstract] AND system[Title/Abstract])) OR (sympathetic[Title/Abstract] AND function[Title/Abstract])) OR (adrenergic[Title/Abstract] AND stimulation[Title/Abstract])) OR (adrenergic[Title/Abstract] AND system[Title/Abstract]))) OR ((((((((MIBG[Title/Abstract] OR nuclear[Title/Abstract]) OR Scintigraphy[Title/Abstract]) OR PET[Title/Abstract]) OR SPECT[Title/Abstract]) OR metaiodobenzylguanidine[Title/Abstract]) OR Iobenguane[Title/Abstract]) OR Positron emission tomography[Title/Abstract]) OR Single-photon emission computed tomography[Title/Abstract]))

**EMBASE**

lv:ab,ti AND end:ab,ti AND diastolic:ab,ti AND volume:ab,ti OR (lv:ab,ti AND end:ab,ti AND diastolic:ab,ti AND pressure:ab,ti) OR (left:ab,ti AND ventricle:ab,ti AND end:ab,ti AND diastolic:ab,ti AND pressure:ab,ti) OR lvedp:ab,ti OR (left:ab,ti AND ventricle:ab,ti AND end:ab,ti AND diastolic:ab,ti AND volume:ab,ti) OR (lv:ab,ti AND wall:ab,ti AND thickness:ab,ti) OR (left:ab,ti AND atrial:ab,ti AND volume:ab,ti) OR (left:ab,ti AND ventricle:ab,ti AND mass:ab,ti) OR (left:ab,ti AND ventricle:ab,ti AND volume:ab,ti) OR (lv:ab,ti AND mass:ab,ti) OR (lv:ab,ti AND volume:ab,ti) OR (la:ab,ti AND volume:ab,ti) OR lvedv:ab,ti OR (heart:ab,ti AND ejection:ab,ti AND fraction:ab,ti) OR diastole:ab,ti OR (hypertrophy:ab,ti AND left:ab,ti AND ventricle:ab,ti) OR (hypertrophy:ab,ti AND left:ab,ti AND ventricular:ab,ti) OR hfnef:ab,ti OR hfpef:ab,ti OR (diastolic:ab,ti AND heart:ab,ti AND failure:ab,ti) OR (heart:ab,ti AND failure:ab,ti AND normal:ab,ti AND ejection:ab,ti AND fraction:ab,ti) OR (heart:ab,ti AND failure:ab,ti AND normal:ab,ti AND (lvef:ab,ti OR ef:ab,ti)) OR (heart:ab,ti AND failure:ab,ti AND ejection:ab,ti AND fraction:ab,ti AND preserved:ab,ti) OR (heart:ab,ti AND failure:ab,ti AND preserved:ab,ti AND (lvef:ab,ti OR ef:ab,ti)) OR (normal:ab,ti OR preserved:ab,ti AND (ventricle:ab,ti OR ventricular:ab,ti) AND heart:ab,ti AND failure:ab,ti AND function:ab,ti) OR (diastolic:ab,ti AND dysfunction:ab,ti) OR (diastolic:ab,ti AND heart:ab,ti AND disease:ab,ti) AND [embase]/lim

AND

sympathetic:ab,ti AND nerve:ab,ti AND system:ab,ti OR (sympathic:ab,ti AND nerve:ab,ti AND system:ab,ti) OR (sympathetic AND nervous:ab,ti AND system:ab,ti) OR (sympathetic:ab,ti AND activity:ab,ti) OR (sympathic:ab,ti AND activity:ab,ti) OR (sympathetic:ab,ti AND nerve:ab,ti AND activity:ab,ti) OR sympathicus:ab,ti OR (orthosympathetic:ab,ti AND activity:ab,ti) OR (orthosympathetic:ab,ti AND nervous:ab,ti AND system:ab,ti) OR (orthosympathetic:ab,ti AND nerve:ab,ti AND system:ab,ti) OR (sympathetic:ab,ti AND (nerve:ab,ti OR nerves:ab,ti)) OR sns:ab,ti OR norepinephrine:ab,ti OR noradrenaline:ab,ti OR noradrenalin:ab,ti OR adrenalin:ab,ti OR adrenaline:ab,ti OR epinephrine:ab,ti OR catecholamine:ab,ti OR catecholamines:ab,ti OR isoproterenol:ab,ti OR iso:ab,ti OR (reactive:ab,ti AND oxygen:ab,ti AND species:ab,ti) OR microneurography:ab,ti OR peroneal AND nerve:ab,ti OR microneurographic:ab,ti OR noradrenergic:ab,ti OR (autonomic:ab,ti AND stimulation:ab,ti) OR (autonomic:ab,ti AND system:ab,ti) OR (sympathetic:ab,ti AND function:ab,ti) OR (adrenergic:ab,ti AND stimulation:ab,ti) OR (adrenergic:ab,ti AND system:ab,ti) OR mibg:ab,ti OR nuclear:ab,ti OR scintigraphy:ab,ti OR pet:ab,ti OR spect:ab,ti OR metaiodobenzylguanidine:ab,ti OR iobenguane:ab,ti OR (positron:ab,ti AND emission:ab,ti AND tomography:ab,ti) OR ('single photon':ab,ti AND emission:ab,ti AND computed:ab,ti AND tomography:ab,ti) AND [embase]/lim

**COCHRANE**

#1 (hfnef):ti,ab;kw or (hfpef):ti,ab,kw or (diastolic heart failure):ti,ab,kw or (heart failure preserved ejection fraction): ti,ab;kw or (heart failure normal ejection fraction):ti,ab;kw

#2 (sympathetic nerve): ti,ab;kw or (sns): ti,ab;kw or (sympathetic nervous): ti,ab;kw or (sympathetic activity): ti,ab;kw or (orthosympathetic activity): ti,ab;kw

#3: #1 AND #2
