# Supplementary material for: A Systematic Review Concerning the Relation between the Sympathetic Nervous System and Heart Failure with Preserved Left Ventricular Ejection Fraction
Source: PLoS One. 2015 Feb 6;10(2):e0117332. doi: 10.1371/journal.pone.0117332 (PMC4319815; doi:10.1371/journal.pone.0117332)
Supplement: S2 Appendix — (DOCX) [file pone.0117332.s003.docx]

**Appendix S2. Summary of included studies in the review**

**Animal studies**

| **Author, year** | **title** | **Species, n** | **Study investigation** | **Main findings** |
| --- | --- | --- | --- | --- |
| **Krishnamurthy, 2007** | (beta)1 integrins modulate (beta)-adrenergic receptor-stimulated cardiac myocyte apoptosis and myocardial remodeling) | Mice,  1.WT-placebo (n=5)  2. Heterozygous knockout mice for β1 integrins -placebo (n=5)  3.WT receiving ISO 0.4 mg/kg/day (n=5)  4. Heterozygous knockout mice for β1 integrins receiving ISO 0.4 mg/kg/day (n=5) | Does a decrease of ß1-integrins cause a significant increased expression and activity of MMP-2 and eventually heart failure? ß1-integrin knockout and wild mice treated with ISO or placebo were evaluated by echocardiography and LV pressure volume-loops. | ß-adrenergic stimulation increased cardiac hypertrophy, apoptosis, and fibrosis in both WT and ß1 KO-mice. However, the increase in fibrosis and hypertrophy was higher in the WT-iso group, whereas the increase in myocyte apoptosis was higher in the ß1 knockout-iso group. EF increased in WT animals and decreased in ß1 knockout mice. |
| **LaCroix, 2008** | Deficiency of M2 muscarinic acetylcholine receptors increases susceptibility of ventricular function to chronic adrenergic stress | Mice, 20  1.M2-AChR KO mice receiving 14-56 ng/kg ISO i.v. (n=5)  2.M2-AChR KO mice receiving placebo (n=5)  3.WT mice receiving 14-56 ng/kg ISO i.v. (n=5)  4. WT receiving placebo (n=5) | Does a decrease of parasympathetic activity (by lack of M2 muscarinic acetylcholine receptors) influence MMP-2 and MMP-9 activity? Mice deficient of M_2_- muscarinic acetylcholine receptor gene (M_2_-AChR KO) were compared to wild type mice. The effect of ISO or placebo on cardiac function was evaluated using pressure volume-loops and activity of MMP. | In M_2_-AChR KO-mice, the activities of both MMP-2 and MMP-9 were significantly increased after stimulation with ISO. In the M_2_-AChR KO mice without ß-adrenergic stimulation, this increase in MMP was not seen. Authors concluded that M_2_-AChR-mediated parasympathetic action may play a protective role in cardiac ventricular function |
| **Brooks, 2009** | Isoproterenol-induced myocardial injury and diastolic dysfunction in mice: structural and functional correlates | Mice,   1. 100 mg/kg ISO s.c. once daily for 5 days (n=15) 2. placebo s.c. for 5 days (n=15) | What are the functional consequences of structural changes induced by ISO?  Mice were injected with ISO once daily for 5 days and evaluated 10-14days later with haemodynamic and morphometric investigations. | In mice treated with ISO, a significant increase in LV diastolic pressure-volume relationship and myocardial fibrosis is observed without significant impairment of systolic function.  Treatment with ISO leads to DD in mice. |
| **Ma, 2011** | Distinct actions of intermittent and sustained beta-adrenoceptor stimulation on cardiac remodeling | Mice, 40   1. Placebo s.c. (n=8) 2. 5 mg/kg/day ISO s.c. (n=8) 3. Placebo pump (n=8) 4. 5 mg/kg/day ISO pump (n=16) | Do different modes of ß-adrenergic stimulation have differential actions on cardiac remodeling? The effects of intermittent versus sustained administration of ISO on cardiac remodeling and function in mice were studied by echocardiography, pressure volume-loops and histology. | Both intermittent and sustained ß-adrenergic stimulation caused cardiac hypertrophy to a similar extent. However, intermittent ß-adrenergic stimulation led to more severe cardiac fibrosis and cardiac dysfunction compared with sustained ß-adrenergic stimulation. The observed detrimental effects on diastolic and systolic function were similar. |
| **Grimm, 1998** | Development of heart failure following isoproterenol administration in the rat: role of the renin-angiotensin system | Rats,   1. Placebo (n=42) 2. 50 mg/ kg ISO (n=10) 3. 100 mg/kg ISO (n=10) 4. 150 mg/kg ISO (n=10) 5. 200 mg/kg ISO (n=10) 6. 250 mg/kg ISO (n=10) 7. 300 mg/kg ISO (n=10) 8. 150 mg/kg ISO s.c. and placebo (n=70) 9. 150 mg/kg ISO s.c. and ramipril 10mg/kg/day (n=34) | Does cardiomyocyte necrosis and interstitial fibrosis induced by catecholamines lead to development of HF? What dosage induces significant myocardial damage with acceptable survival rate? Rats were injected by placebo, Iso or Iso in combination with ramipril. The effects were evaluated by echocardiography, laboratory assessment and histology. | A single injection of ISO up to 150mg/kg induces a syndrome that displays numerous typical characteristics of mild heart failure and diastolic dysfunction.  Dosages >150 mg/kg resulted in death rates of 50-90% in the first 24 hours. |
| **Yoshikawa, 2012** | Increased Passive Stiffness of Cardiomyocytes in the Transverse Direction and Residual Actin and Myosin Cross-Bridge Formation in Hypertrophied Rat Hearts Induced by Chronic beta-Adrenergic Stimulation | Rats, 20  1.ISO 2.4 mg/kg/day for 7 days (n=10)  2.Placebo for 7 days (n=10) | What is the passive cardiomyocyte stiffness of hypertrophied hearts in the transverse direction? Secondly, is the stiffness influenced by actin-myosin cross-bridge formation? The effects were evaluated by echocardiography and histology. | Administration of isoproterenol caused significant LV hypertrophy and fibrosis, and decreased LV diastolic function such as decreased e’ and increased E/e’. |

DD: diastolic dysfunction; LV: left ventricle; ISO: isoproterenol; s.c.: subcutaneous injection; WT: wild type; MMP: metalloproteinase; M_2_-AChR KO: Mice deficient of M_2_- muscarinic acetylcholine receptor gene; e’: early diastolic mitral annular velocity; E/e’: the ratio of peak early transmitral ventricular filling velocity to early diastolic tissue Doppler velocity

**Human studies**

| **Author, year** | **Title** | **Population** | **Study investigation** | **Main findings** |
| --- | --- | --- | --- | --- |
| **Nixdorff, 1997** | Beta-adrenergic stimulation enhances left ventricular diastolic performance in normal subjects | 10 healthy volunteers  1. Treated with ISO, dosages were 0.1, 0.2, 0.4, 0.75, and 1.5 ug/min for a period of 15 minutes for each step (n=?)  2. Treated with placebo (n=?) | What is the effect of beta-adrenergic stimulation on flow characteristics of LV diastolic filling assessed by echocardiography? | Adrenergic stimulation leads to enhancement of diastolic performance as reflected by an increase of V_maxE_, V_maxA_, VTI E, VTI A, and a shortening of T_dec_. |
| **Hirono, 2001** | Left ventricular diastolic dysfunction in patients with bronchial asthma with long-term oral beta2-adrenoceptor agonists | 1. Patients with asthma and regular long-term use of oral β2- adrenergic stimulation (n=26/74)  2. Patients with asthma without β2- adrenergic stimulation (n=22/69)  3. Healthy controls (n=21) | What are the long-term effects of β2-adrenergic stimulation on cardiac function? Patients with asthma and long-term use of β2- adrenergic stimulation were compared to asthmatic patients not using β2- adrenergic stimulation. | After long-term intake of oral β2- adrenergic stimulation, patients with bronchial asthma showed retrospectively and prospectively to have left ventricular diastolic dysfunction on echocardiography. Cessation of β2- adrenergic stimulation returned diastolic function to normal. |
| **Vinch, 2003** | Usefulness of clinical variables, echocardiography, and levels of brain natriuretic peptide and norepinephrine to distinguish systolic and diastolic causes of acute heart failure | 1. Patients with HFPEF (n=14)  2. Patients with HFREF (n=16) | Can clinical evaluation, echocardiography, or neurohumoral profiling distinguish HFPEF from HFREF in HF patients presented to the emergency department? Do changes in neurohumoral parameters parallel improvement in clinical status? | Plasma NE concentrations were significantly higher in HF patients compared to controls, but could not distinguish HFPEF from HFREF. Nor did they change significantly after initial treatment. |
| **Arora, 2004** | Heart rate variability and diastolic heart failure | 1. Patients with HFPEF (n=19)  2. Patients with HFREF (n=9)  3. Controls (n=9) | Does HFPEF lead to changes in sympathetic-parasympathetic balance? | Patients with HFPEF exhibit a reduction in many measures of HRV. HRV parameters in patients with HFPEF were not as profoundly reduced as in patients with HFREF. |
| **Piccirillo, 2006** | Autonomic cardiovascular control and diastolic dysfunction in hypertensive subjects | 1. Hypertensives with normal LV filling (n=30) 2. Hypertensives with abnormal LV filling (n=30) 3. Controls (n=29) | Is there a difference in autonomic nerve control between hypertensive patients with and without diastolic dysfunction? Heart rate variability was used for assessment of sympathetic activity. | Hypertensives with diastolic dysfunction have higher sympathetic and lower vagal modulation of the sinus node during HRV compared with hypertensives with a normal diastolic function and normotensive controls. |
| **Sugiura, 2006** | The relationship between variables of 123-I-metaiodobenzylguanidine cardiac imaging and clinical status of the patients with diastolic heart failure | Patients with HFPEF (n=34) | What is the relationship between cardiac sympathetic nerve activity and HFPEF? | Cardiac sympathetic nerve activity increases proportionally with severity of diastolic heart failure |
| **Tsuchida, 2007** | Relationship between plasma norepinephrine at peak exercise and 123I-MIBG imaging of the heart and lower limbs in heart failure | 1. Patients with LVEF ≥ 45% (n=8)  2. Patients with LVEF < 45% (n=12) | What is the relation between plasma norepinephrine concentration and the function of sympathetic nerve terminals distributed to the heart and the right lower limb in patients with heart failure with preserved or impaired LV function? | Plasma norepinephrine was correlated to MIBG heart/brain ratio in HFPEF patients and to MIBG limb/brain ratio in HFREF patients. In HFPEF-patients, plasma NE originates mainly in the myocardium and in HFREF in the skeletal muscles. It is unclear whether the origin of norepinephrine in healthy subjects is similar to that in patients with preserved or low LVEF. |
| **Grassi, 2009** | Sympathetic and baroreflex cardiovascular control in hypertension- related left ventricular dysfunction | 1. Hypertensives with DD (n= 17)  2. Hypertensives without DD (n=20)  3. Normotensive controls (n=20) | Is there a sympathetic overdrive in left ventricular diastolic dysfunction? | MSNA values were markedly and significantly greater in hypertensive than normotensive patients and in hypertensive patients with than without diastolic dysfunction. |
| **deSouza, 2013** | High muscle sympathetic nerve activity is associated with left ventricular dysfunction in treated hypertensive patients | 1.Normotensive controls (n=14)  2.Hypertensives with normal diastolic function (n=15)  3.Hypertensives with impaired relaxation DD (n=15)  4.Hypertensives with pseudonormal or restrictive classes of DD (n=15) | Is sympathetic activity greater in hypertensive patients with diastolic dysfunction independent of BP control?  Do different classes of DD have different effects of sympathetic activity?  Echocardiography was used to distinguish the different types of DD. | 1. Hypertensive patients with diastolic dysfunction have a higher MSNA than hypertensive patients without DD.  2. The stage of DD shows no effect on sympathetic activity. |

HFPEF: heart failure with preserved ejection fraction; HFREF: heart failure with reduced ejection fraction; DD: diastolic dysfunction; HRV: heart rate variability; ISO: isoproterenol; LV: left ventricle; NE: norepinephrine; LVEF: left ventricular ejection fraction.
